# Metabolic Food Waste and Ecological Impact of Obesity in FAO World's Region

**DOI:** 10.3389/fnut.2019.00126

**Published:** 2019-08-23

**Authors:** Elisabetta Toti, Carla Di Mattia, Mauro Serafini

**Affiliations:** ^1^Nutritional Quality and Sustainability in Preventing the Metabolic Stress, Centre for Food and Nutrition, Council for Agricultural Research and Economics, Rome, Italy; ^2^Functional Foods and Metabolic Stress Prevention Laboratory, Faculty of Bioscience and Technology for Food, Agriculture and Environment, University of Teramo, Teramo, Italy

**Keywords:** sustainable nutrition, obesity, metabolic food waste, functional diet, ecological footprints, food balance sheets, human, animal products

## Abstract

Obesity represents a titanic cost for the world's health systems but also a substantial ecological cost to the environment. High energy foods have been shown to be the major contributor to Greenhouse Gas (GHG) emissions, challenging the diet-environment-health triangle. The waste of resources and the unnecessary ecological cost due to an excessive consumption of foods leading to obesity have been ignored so far. Metabolic Food Waste [MFW_(kg of food)_] corresponds to the amount of food leading to Excess Body Fat (EBF) and its impact on the environment, expressed as carbon [MFW_(kgCO_2_eq)_], water [MFW_(×10 L)_] and land footprint [MFW_(×10 m^2^)_]. We aim to estimate the MFW_(kg of food)_ in the seven FAO regions, Europe (EU), North America and Oceania (NAO), Latin America (LA), Sub-Saharan Africa (SSA), Industrialized Asia (IA), North Africa, West and Central Asia (NAWCA) and South and Southeast Asia (SSEA), and evaluate its impact on ecological footprints. The overall impact of MFW_(tons of food)_ in the world corresponds to 140.7 million tons associated to overweight and obesity. Between the different regions, EU is responsible of the greatest amount of MFW_(tons of food)_ volume (39.2 million tons), followed by NAO (32.5 million tons). In terms of ecological impact, EU and NAO displayed the highest values for all three MFW footprints, about 14 times more than SSA. We provide evidence of the enormous amount of food lost through obesity and its ecological impact. Reducing metabolic food waste associated with obesity will contribute in reducing the ecological impact of unbalanced dietary patterns through an improvement of human health.

## Introduction

Obesity is a chronic metabolic disorder with a complex etiology representing a remarkable risk factor for the onset of different chronic diseases, such as cardiovascular disease (CVD), diabetes type 2 and cancers, responsible for 60% of deaths worldwide (1). Over the past decade, the obesity burden in western and in developing countries has more than doubled: the WHO estimates that more than 1.9 billion adults and 41 million children under the age of five are overweight or obese (2). Between the different causes, obesity is basically due to an excess consumption of calories over time being turned into body fat, with abdominal fat as a reliable index of negative impacts on health (3). However, obesity condition, other than being an excessive fat deposit, is characterized by excessive and uncontrolled cytokines, free radicals and adhesion molecule production, a condition defined as “low-grade chronic inflammation” associated with the development of degenerative diseases (4, 5). Moreover, due to the uncontrolled high intake of nutritionally unbalanced meals, characterized by elevated caloric, sugars, and fat consumption, obesity condition is also associated with continuous postprandial metabolic stress, characterized by a deep raising of CVD risk factors such as blood pressure, insulin resistance, LDL oxidation, triglycerides and high glucose blood levels.

Consumption of food that is constantly above the recommended calorie requirements by a growing number of people not only represents a risk to health, but it also puts more pressure on natural resources and on the environment (1), including overeating among food waste (6). The calculated progressive increase of food waste suggests that the worldwide obesity epidemic has been the result of a “push effect” of increased food availability and marketing. Since 1974 the energy content of diet has increased 50%, reaching more than 1,400 kcal per person per day or 150 trillion per year (7). In this view, obesity condition represents a considerable cost for the environment (8). Recently, we introduced a new index to calculate the ecological impact of obesity, Metabolic Food Waste [MFW_(kg of food)_], corresponding to the amount of food leading to Excess Body Fat (EBF) and its impact on the environment expressed as carbon [MFW_(kgCO_2_eq)_], water [MFW_(×10 L)_], and land footprint [MFW_(×10 m^2^)_] (8). We estimated MFW_(kg of food)_ of 63.1 and 127.2 kg per capita in an observational study on 60 overweight and obese subjects, and of 2.081 million kg of food for the Italian population, with the highest contributor from animal products in terms of carbon emissions, water consumption and land use. We claim that increased population adiposity, because of its contribution to climate change from unnecessary food consumption, should be recognized, in addition to being a health and sociological issue, as an environmental problem.

Therefore, the aim of our work is to estimate the MFW_(kg of food)_ in the seven FAO regions and evaluate its impact on climate, water, and land footprints for food commodity groups in order to provide a global picture of the phenomenon.

## Materials and Methods

Prevalence of overweight and obesity at a global level from the WHO Global Database on Body Mass Index (BMI) relative to the year 2017 (9) has been used in combination with population data (10) to assess the number of individuals belonging to BMI categories of overweight and obese subjects. In order to assess global Excess Body Fat (EBF) in terms of mass (kg), the difference between average body weight at a national level and “*ideal body weight*,” given by BMI inverse function, has been calculated. The “*ideal BMI”* estimated by the midpoint between lowest and highest values using WHO cut-off points for BMI resulted to be 21.7 kg/m^2^ for normal the weight category [(18.5 + 24.9)/2 = 21.7]; while average body height by country was obtained from DHS projects (11) on all completed population-based surveys, the national statistical databases were also used for missing data.

The attained EBF was multiplied for the energy content of 1 kg of body fat (32.2 MJ) to reach the energy from EBF and distributed among the different foods according to their percentage contribution to total energy intake. An estimation of food items contributing to the EBF has been calculated associating energy from EBF with kilocalories from food, determined from the Food Balance Sheets (FBS) in FAOSTAT (12). The percent energy contribution of each food item at the national level was multiplied with the total amount of energy from EBF for each specific country to achieve the amount of energy of each food item contributing to the EBF, with the value translated into amount of food wasted through overweight/obesity [MFW_(tons of food)_]. Food waste was based on total production through FBS food items grouped into nine main groups: dairy products/milk/eggs, starchy roots, alcoholic beverages, cereals, meat/offals, sugar and sweeteners, fish/seafood, added fats, and pulses. The acquired data allowed us to provide a worldwide estimation of the impact of over-nutrition on planet health through the new index MFW_(tons of food)_ and its impact on the environment as carbon [MFW_(kgCO_2_eq)_], water [MFW_(m^3^ water)_], and land footprint [MFW_(m^2^ land)_] (13, 14). Complete information has been reached for 86 countries, categorized in the seven FAO world regions: Europe (EU), North America and Oceania (NAO), Latin America (LA), Sub-Saharan Africa (SSA), Industrialized Asia (IA), North Africa, West and Central Asia (NAWCA), and South and Southeast Asia (SSEA).

## Results

As displayed in Table 1, the overall impact of MFW_(tons of food)_ in the world correspond to 140.7 million tons of food waste associated with overweight and obesity. Between the different regions, Europe (EU) is responsible of the greatest amount of MFW_(tons of food)_ volume (39.2 million tons), followed by North America and Oceania (32.5 million tons) (NAO) and Latin America (20 million tons) (LA), while the lowest extent of MFW was recorded in Sub-Saharan Africa (SSA) with 5 million tons as described in Table 1. It is interesting to note that the highest percentage of MFW_(tons of food)_ in NAO came from obese people (67.7%), however, EU with a similar percentage between OWand OB accounted for the highest volume of MFW.

**Table 1:** Metabolic Food Waste [MFW_(kg of food)_] corresponding to Excess Body Fat by BMI categories (OW, Overweight; OB, Obesity).

	**MFW_**(tons of food)**_**	**% From**	
		**OW**	**OB**
EU	39,201,410,847	48.6	51.4
NAO	32,465,755,707	32.3	67.7
LA	20,022,343,875	44.9	55.1
IA	17,190,412,965	71.7	28.3
NAWCA	14,595,049,642	35.7	64.3
SSEA	12,181,476,616	59.6	40.4
SSA	5,079,066,441	47.4	52.6
Total worldwide	140,735,516,093		

*EU, Europe; NAO, North America and Oceania; LA, Latin America; IA, Industrialized Asia; NAWCA, North Africa, West and Central Asia; SSEA, South and Southeast Asia; SSA, Sub-Saharan Africa*.

**Figure 1:**
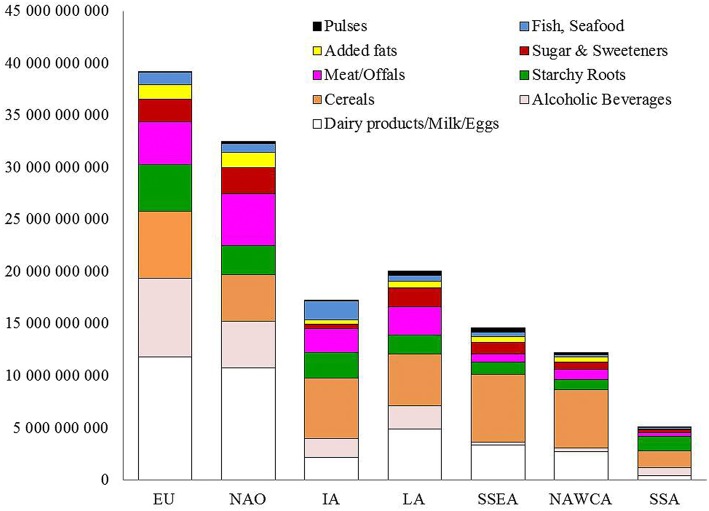
Metabolic Food Waste corresponding to excess body fat from FBS commodities in overweight and obese population expressed as amount of food [MFW_(kg of food)_]. EU, Europe; NAO, NorthAmerica and Oceania; LA, Latin America; IA, Industrialized Asia; NAWCA, North Africa, West and Central Asia; SSEA, South and Southeast Asia; SSA, Sub-Saharan Africa.

As described in Figure 1, dairy products/milk/eggs, were the highest contributor to MFW_(tons of food)_ in the EU (about 12 million tons corresponding to 30.2%) and NAO (33.1%), followed by alcoholic beverages (19.1%) and cereals (16.4%) in EU,meat/offals (15.2%) and alcoholic beverages (13.9%) in NAO. Cereals and dairy products were the two largest contributors to MFW_(tons of food)_ in LA (24.8 and 24.4%, respectively), SSEA (44.6 and 22.8%) and NAWCA (46.0 and 22.2%), while in IA and SSA the highest impact came from cereals (33.8 and 31.5%, respectively) and starchy roots (14.2 and 28.0%).

In terms of ecological impact, EU and NAO displayed the highest values for all three MFW footprints (water, land, carbon), about 14 times more than SSA that, as for the MFW_(tons of food)_, is the area with lowest ecological impact (Table 2). The water footprint for EU and NAO was similar but almost double with respect to LA and 10 times more with respect to the ones with lowest impact (SSA). In terms of GHG emissions, EU was in first place followed by NAO and LA, also here the footprint of EU was almost 10 times more than SSA. Regarding land use, EU was the first followed by NAO and LA, with a land use of about 15 times higher than SSA.

**Table 2:** Metabolic Food Waste (MFW) expressed as water (millions m^3^), GHG emissions (millions kg/CO_2_eq), land (millions m^2^).

	**Water (millions m^**3**^)**	**GHGs (millions kg/CO_**2**_eq)**	**Land (millions m^**2**^)**
EU	93,926,391	66,477,365	1,085,945,294
NAO	92,446,368	58,419,124	1,034,147,016
IA	36,982,188	31,403,573	416,548,943
LA	50,769,141	34,343,457	531,386,727
NAWCA	28,631,298	18,885,007	289,849,101
SSEA	32,220,628	22,626,903	355,688,254
SSA	8,339,655	7,253,364	71,353,289
Total worldwide	343,315,669	239,408,793	3,784,918,624

*EU, Europe; NAO, North America and Oceania; LA, Latin America; IA, Industrialized Asia; NAWCA, North Africa, West and Central Asia; SSEA, South and Southeast Asia; SSA, Sub-Saharan Africa*.

**Figure 2:**
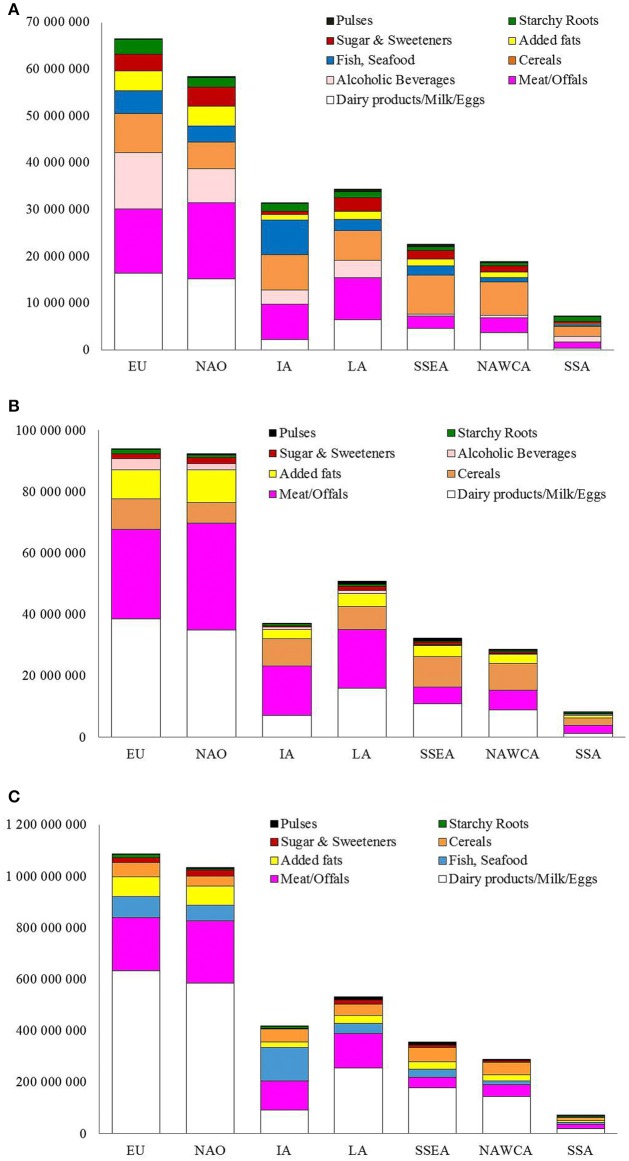
Metabolic Food Waste corresponding to Excess Body Fat from FBS commodities in overweight and obese population expressed as **(A)** GHG emission, MFW_(millions kgCO_2_eq)_; **(B)** water consumed, MFW_(millions m^3^)_; and **(C)** land used, MFW_(millions m^2^)_. EU, Europe; NAO, North America and Oceania; LA, Latin America; IA, Industrialized Asia; NAWCA, North Africa, West and Central Asia; SSEA, South and Southeast Asia; SSA, Sub-Saharan Africa.

Figure 2A shows the contribution of commodities to total GHG emissions by geographical area. Dairy products/milk/eggs and meat/offals have the highest values in EU and NAO, two geographical areas where overweight and obesity are at epidemic proportions, followed by alcoholic beverages. Meat/offals are the highest contributor in IA followed by cereals and fish/seafood, while in LA, SSEA and NAWCA, the first three places were occupied by cereals, dairy products/milk/eggs and meat/offals whereas in SSA cereals were the highest contributor.

Figure 2B shows total water footprint (as millions m^3^) from MFW in the seven regions: dairy products/milk/eggs and meat/offals as a whole contribute to more than 70% in EU and NAO. In IA meat/offals was in first place (43.7%), followed by cereals (23.8%). In LA, SSEA, and NAWCA dairy products/milk/eggs and cereals were the two highest contributor to MFW water footprint.

Animal commodities, dairy products/milk/eggs, meat/offals and fish, were the highest contributors to the MFW_(millions m^2^)_ for land footprint with about 80% of the contribution in EU and NAO, as displayed in Figure 2C. Specifically, dairy products/milk/eggs and meat/offals were at the first two places for all regions, with the only exception of IA where the first contributor to land use was fish/seafood (31.0% against <10% in the other geographical areas). Data on MFW for land footprint does not include alcoholic beverages because they were not available in the footprints database.

## Discussion

Obesity is a social challenge that is rising in almost all countries with detrimental effects for human and planet health. In this work we showed that the overall impact of MFW_(tons of food)_ associated with overweight and obesity in the world is 140.7 million tons of food waste, with EU and NAO with the highest ecological impact for water, land and carbon footprints. We proved that obesity burden represents a significant additional increment to the already high global statistics on food wastage causing an unsustainable ecological cost for the planet. However, if we consider that increase in body fat is the result of a long period of reduced physical activity as well as a diet rich in energy-dense food and an intake of calories much higher than metabolic needs, our figuresmight have underestimated the amount of food wasted through the increase in body fat. In order to properly assess the amount of food wasted through obesity, MFW should be calculated in long-term observational/epidemiological studies together with measurements of metabolic parameters.

Our results show that industrialized regions such as EU and NAO display a much higher ecological impact, associated with excess body fat, compared to developing regions like NAWCA and SSA. Our results mirror the main findings from a previous FAO report (15) showing higher values of food waste in the EU and NAO compared to non-industrialized countries, highlighting the responsibility of high-income countries in food waste. It is important to study approaches to address behavior changes in the developed regions, raising the consciousness on the importance of reducing the waste of food and to prefer dietary patterns suited for individual energy needs, to avoid a further increase of the environmental and health impacts associated with unbalanced western dietary habits.

Between the different commodities, meat/offals and dairy products/milk/eggs were shown to be major contributors to MFW_(tons of food)_ and the ecological footprints of EU and NAO and in almost all FAO areas, with few exceptions like cereals in SSEA and NAWCA (Figure 2A). It has been widely shown that animal products have much higher ecological costs compared to plant-based foods (16); however, the role of animal products in people's diet must be carefully considered within the context of each region without neglecting the higher nutritional values of these products for populations suffering from malnutrition. As stated from the EAT-Lancet Commission (17), meat consumption in the world is about 288% times higher than the amount suggested for a balanced and healthy diet. Obviously, reducing meat, dairy, milk, and egg consumption in industrialized countries must be a priority to reduce the ecological burden of animal products.

Finally, it shall be emphasized that our data provide important information because they quantify the effects of over-nutrition on the GHGs emissions, water and land use related to the availability of food at worldwide level, through food balance sheets. However, it must be considered that our results are based on national availability of the main food commodities, providing a valuable insight into diets but not corresponding to average food intake or average food consumption.

Strategies of disease prevention should also be focused on overweight people, representing an excellent target to avoid the development of obesity condition and to minimize the ecological cost of excessive food intake. Overall, we think that it is extremely important to raise awareness in the population at large of the impressive waste of food and natural resources associated with overweight and obesity. At a public health level, it should be highly desirable to develop a specific campaign for obesity prevention focused on increasing physical activity as well as in reducing metabolic food waste through a wise and ethical approach to food consumption.

## Conclusion

We provide evidence, at world level, of the enormous amount of food lost through obesity and its ecological impact. As expected, animal products were the highest contributor to MFW; however, large epidemiological studies are needed in order to clearly identify major dietary contributors to MFW in humans. Reducing metabolic food waste associated with obesity will contribute to reducing the ecological impact of unbalanced dietary patterns through an improvement of human health.

## Data Availability

The datasets generated for this study are available on request to the corresponding author.

## Author Contributions

MS designed the experiment. ET performed data analyses. MS, ET, and CD contributed to data interpretation and manuscript drafting.

### Conflict of Interest Statement

The authors declare that the research was conducted in the absence of any commercial or financial relationships that could be construed as a potential conflict of interest.

## Abbreviations

GHGs: Greenhouse Gases
MFW: Metabolic Food Waste
EBF: Excess Body Fat
EU: Europe
NAO: North America and Oceania
LA: Latin America
SSA: Sub-Saharan Africa
IA: Industrialized Asia
NAWCA, North Africa: West and Central Asia
SSEA: South and Southeast Asia
CVD: Cardiovascular disease
WHO: World Health Organization
BMI: Body Mass Index
FBS: Food Balance Sheets.
