# Corrigendum: Metabolic Food Waste and Ecological Impact of Obesity in FAO World's Region

**DOI:** 10.3389/fnut.2019.00160

**Published:** 2019-10-11

**Authors:** Elisabetta Toti, Carla Di Mattia, Mauro Serafini

**Affiliations:** ^1^Nutritional Quality and Sustainability in Preventing the Metabolic Stress, Centre for Food and Nutrition, Council for Agricultural Research and Economics, Rome, Italy; ^2^Functional Foods and Metabolic Stress Prevention Laboratory, Faculty of Bioscience and Technology for Food, Agriculture and Environment, University of Teramo, Teramo, Italy

**Keywords:** sustainable nutrition, obesity, metabolic food waste, functional diet, ecological footprints, food balance sheets, human, animal products

In the original article, there was a mistake in the legend for **Figure 2** as published.

It was not written as millions of kg in MFW_(kgCO_2_eq)_.

The correct legend appears below.

**Figure 2**. Metabolic Food Waste corresponding to Excess Body Fat from FBS commodities in overweight and obese population expressed as **(A)** GHG emission, MFW_(millions kgCO_2_eq)_; **(B)** water consumed, MFW_(millions m^3^)_; and **(C)** land used, MFW_(millions m^2^)_. EU, Europe; NAO, North America and Oceania; LA, Latin America; IA, Industrialized Asia; NAWCA, North Africa, West and Central Asia; SSEA, South and Southeast Asia; SSA, Sub-Saharan Africa.

In the original article, there was an error in **Table 1** as published. It was written MFW_(tons of food)_ instead of MFW_(kg of food)_.

The correct title appears below.

**Table 1**. Metabolic Food Waste [MFW_(**kg of food**)_] corresponding to Excess Body Fat by BMI categories (OW, Overweight; OB, Obesity).

In the original article, there was an error. The values of MFW_(tons of food)_ were expressed in gigatons instead of millions of tons.

Corrections have been made to the **Abstract:**

The overall impact of MFW_(tons of food)_ in the world corresponds to 140.7 **million tons** associated to overweight and obesity. Between the different regions, EU is responsible of the greatest amount of MFW_(tons of food)_ volume (39.2 **million tons**), followed by NAO (32.5 **million tons**).

Corrections have been made to **Results, Paragraph 1:**

As displayed in Table 1, the overall impact of MFW_(tons of food)_ in the world correspond to 140.7 **million tons** of food waste associated with overweight and obesity. Between the different regions, Europe (EU) is responsible of the greatest amount of MFW_(tons of food)_ volume (39.2 **million tons**), followed by North America and Oceania (32.5 **million tons**) (NAO) and Latin America (20 **million tons**) (LA), while the lowest extent of MFW was recorded in Sub-Saharan Africa (SSA) with 5 **million tons** as described in Table 1.

As described in Figure 1, dairy products/milk/eggs, were the highest contributor to MFW_(tons of food)_ in the EU (about 12 **million tons** corresponding to 30.2%)…….

A correction has been made to **Discussion, Paragraph 1:**

In this work we showed that the overall impact of MFW_(tons of food)_ associated with overweight and obesity in the world is 140.7 **million tons** of food waste…

The authors apologize for these error and state that this does not change the scientific conclusions of the article in any way. The original article has been updated.

